# Medical Cases, with Occasional Remarks

**Published:** 1818-10-01

**Authors:** Edward Carbutt

**Affiliations:** Member of the Royal Physical Society of Edinburgh, and of the Literary and Philosophical Society of Manchester; one of the Physicians to the Manchester Infirmary and Dispensary; Lunatic Hospital and Asylum; and to the House of Recovery


*Medical Cases, with occasional Remarks.* By Edward Carbutt, M. D. Member of the Royal Physical Society of Edinburgh, and of the Literary and Philosophical Society of Manchester; one of the Physicians to the Manchester Infirmary and Dispensary; Lunatic Hospital and Asylum; and to the House of Recovery.

---

Case 1. *Functional Disease of the Heart, resembling Organic Disease, but ultimately disappearing without Relapse.*

In pages 118 and 119 of the first quarterly number of the Medico-Chirurgical Journal, (July last) an abstract is given of a case of Organic Disease of the Heart, recorded by me in the Annals of Medicine, vol. ii. p. 361. The abstract just mentioned, concludes with the following observation: "We have seen all the above-mentioned symptoms, however, most exquisitely simulated by functional or nervous irritation, and entirely disappear, after every one had pronounced the case desperate and incurable." The present case, which is faithfully, and, I trust, accurately reported, is now presented as a confirmation of the above remark.

October 27, 1817. John Darbey, farrier's apprentice, aged twelve years and a half, was admitted a home-patient* of the Manchester Infirmary and Dispensary.

The symptoms are as follow. Great emaciation of every part of the body; general paleness; face has a puffy appearance. He complains of want of breath, but is able to make a full inspiration. Respirations are twenty-three in a minute, performed by the ribs. A fortnight ago he had much cough with expectoration of mucus tinged with blood. He is able to lie in the recumbent posture. Has violent pain in the situation of the point of the ensiform cartilage. The heart beats on an extended surface, and in a fluttering manner. Pulse 120 in the minute, small, and weak. Tongue foul; thirst great. Has had considerable diarrhœa for some weeks past. Urine high coloured, but in the natural quantity. Skin is rough to the feel; nails and lips are of the natural hues. He sleeps ill, and has

---

* Home patients are such as are seen at their own homes: they are always so ill as to be confined to the house. This explanation seems necessary, as in your abstract you appear to have misunderstood the term. The patient there mentioned, was a home patient.

alarming dreams, terrifying his mother with what he utters during sleep. The mother imputes the complaint to his working at his trade with a large sledge hammer above his strength. It is twelve weeks since he left off working with this hammer, and since that time he has been under the care of a respectable surgeon of this town.

The following was the medicine which I ordered.

℞ Spiritûs ætheris nitrici f ʒvi; Tincturæ opii; Tincturæ digitalis, singulorum f ʒj; Syrupi simplicis; spiritûs lavandulæ compositi, singulorum f ʒij; Aquæ menthæ viridis ℥vjß. Misce: sumatur f ℥ß quater quotidie. Applicetur epigastrio cataplasma domesticum.

28. Symptoms considerably relieved. Pergat.

29. Appears weaker. Pulse 130. Pergat.

30. Symptoms are all remarkably relieved. Passed a quiet night, and slept tolerably. This morning got up from bed, and eat his breakfast with appetite. Breathing is easy. Diarrhœa has abated, and stools are natural. Pergat ut antea.

31. Symptoms continue much milder, but the general emaciation is extreme. Pulse 112; rather stronger. Thirst continues so great that he requires two or three quarts of drink in the day. Skin moist, but still rough. Contin. mistura ut antea.

℞. Potassæ supertartratis ʒij; Syrupi zingiberis f ℥j; Decocti hordei f ℥xxxj. Misce: fiat potus ad libitum sumendus.

Nov. 1. The want of breath has returned to a great degree; other symptoms as before. Pergat.

2. His shortness of breath, as he calls it, is such that he can no longer lie except in the semi-recumbent posture. Tongue clean, and preternaturally red. Thirst continues great; appetite is craving. Has about three evacuations from the bowels daily. Urine is scanty, and indeed almost suppressed. Skin is hot, but not very dry. No œdema. Pergat ut antea.

3. To day I found him up, sitting by the fire side. On my enquiring how he was, he answered, "Quite well." His appetite continues remarkably keen. Continuentur mistura et potus.

6. Still sits up, and eats with appetite. Legs are rather œdematous. Pergat.

10. At present he is surprisingly well. Breathing is perfectly free and easy. Tongue is clean. Has still much thirst. When asked how his appetite was, he laughed and said, "very good." Has two or three healthy looking evacuations daily. Urine natural in kind and quantity

The heart beats in a more natural manner. Has no pain at the epigastrium, as before. Sleeps well, but sweats much at night. The emaciation is not so great as before. Cont. medicamina.

17. Is able to walk out of doors. Bowels quite regular; spirits are remarkably good; thirst, however, continues; pulse 128, weak; passes much urine. Pergat.

December 2. None of the original bad symptoms are now to be observed. He sits up and walks about quite cheerful, and apparently well. Cont. med.

15. Continues so well that he is now an out-patient.* Cont. med.

January 15, 1818. Has continued apparently perfectly well. Has now no palpitation of the heart. Pulse 88, rather weak; breathing quite easy. No œdema; tongue clean; no thirst; appetite keen, though not so voracious as at the time of last report. Bowels quite regular; urine in natural quantity, and of a healthy appearance; has now no remarkable degree of emaciation. Agreeably to my advice, his mother sends him to school instead of hard labour. Omittantur medicamina antea præscripta.

℞. Columbæ radicis contritæ ℈iv; Rhei radicis contritæ ʒß; Extracti taraxaci ℈j; Syrupi zingiberis q. s. e. ad pilulas xxxvj fingendas. Sumantur tres ter quotidie.

February 10. Discharged cured.

July 13. I made inquiry after him to-day, and found that he had had no return whatever of his complaint. His mother's expression was, "that he had had neither ache nor pain since I last saw him."

### REMARKS BY THE EDITORS.

For several years past we have directed our attention to functional and organic diseases of the heart, and notwithstanding the difficulty of always drawing the line of distinction between the two forms of disease, we can assure our professional brethren that our diagnosis has often been greatly assisted by abdominal pressure and thoracic percussion. We earnestly recommend examinations of this kind in all obscure cases. During the last two years we have rarely failed in distinguishing functional from structural lesion of the central organ of the circulation. To Dr. Carbutt our thanks are due. He has the advancement of medical science at heart; and to every man of this kind we are linked by indissoluble chains.

* One who walks up to the Dispensary for advice and medicines.

